# Isolation, Identification, and Bioinformatic Analysis of Antibacterial Proteins and Peptides from Immunized Hemolymph of Red Palm Weevil *Rhynchophorus ferrugineus*

**DOI:** 10.3390/biom11010083

**Published:** 2021-01-11

**Authors:** Stanisław Knutelski, Mona Awad, Natalia Łukasz, Michał Bukowski, Justyna Śmiałek, Piotr Suder, Grzegorz Dubin, Paweł Mak

**Affiliations:** 1 Department of Entomology, Institute of Zoology and Biomedical Research, Jagiellonian University, Gronostajowa 9 St., 30-387 Krakow, Poland; s.knutelski@uj.edu.pl; 2 Department of Economic Entomology and Pesticides, Faculty of Agriculture, Cairo University, Giza 12613, Egypt; mona.awad2003@gmail.com; 3 Department of Analytical Biochemistry, Faculty of Biochemistry, Biophysics and Biotechnology, Jagiellonian University, Gronostajowa 7 St., 30-387 Krakow, Poland; nlukasz.btch@gmail.com (N.Ł.); m.bukowski@uj.edu.pl (M.B.); justyna.smialek@doctoral.uj.edu.pl (J.Ś.); 4 Department of Analytical Chemistry and Biochemistry, Faculty of Materials Sciences and Ceramics, AGH University of Science and Technology, Mickiewicza 30 Ave., 30-059 Krakow, Poland; piotr.suder@agh.edu.pl; 5 Malopolska Centre of Biotechnology, Jagiellonian University, Gronostajowa 7a, 30-387 Kraków, Poland; grzegorz.dubin@uj.edu.pl

**Keywords:** *Rhynchophorus ferrugineus*, antimicrobial proteins/peptides, defensins, cecropins, attacins, pheromone-binding proteins, odorant-binding proteins, bioinformatics

## Abstract

Red palm weevil (*Rhynchophorus ferrugineus* Olivier, 1791, Coleoptera: Curculionidae) is a destructive pest of palms, rapidly extending its native geographical range and causing large economic losses worldwide. The present work describes isolation, identification, and bioinformatic analysis of antibacterial proteins and peptides from the immunized hemolymph of this beetle. In total, 17 different bactericidal or bacteriostatic compounds were isolated via a series of high-pressure liquid chromatography steps, and their partial amino acid sequences were determined by N-terminal sequencing or by mass spectrometry. The bioinformatic analysis of the results facilitated identification and description of corresponding nucleotide coding sequences for each peptide and protein, based on the recently published *R. ferrugineus* transcriptome database. The identified compounds are represented by several well-known bactericidal factors: two peptides similar to defensins, one cecropin-A1-like peptide, and one attacin-B-like protein. Interestingly, we have also identified some unexpected compounds comprising five isoforms of pheromone-binding proteins as well as seven isoforms of odorant-binding proteins. The particular role of these factors in insect response to bacterial infection needs further investigation.

## 1. Introduction

Currently, there is a worldwide rapid increase in the number of pathogenic bacteria with resistance to the array of available antibiotics, which poses a growing threat to human and animal health [1,2]. To overcome this problem, antimicrobial peptides/proteins (AMPs) have come to the forefront as potential antibiotic surrogates with robust killing activity against a wide spectrum of bacterial species, including drug-resistant strains [3]. They exert an antimicrobial effect mainly by disrupting the microbial membrane, which makes microbes unable to easily develop resistance against these compounds [4,5,6]. Additionally, they often display positive immunomodulatory functions, such as modulation of cytokine production, chemotactic activity or promote wound healing [7,8].

Another most important contemporary problem are insect pests of plants that are very difficult to control and cause enormous loss of global crop, forest, and stored products [9,10,11,12]. Insects lack the adaptive immune system; hence, their innate immune mechanisms are particularly efficient [13]. This efficiency together with a wide array of produced AMPs is thought to be one of the biological attributes that can explain the evolutionary success of insects [5,14]. Insect AMPs can be classified into four main groups based on their structure or unique sequences: α-helical peptides (cecropins and moricins), cysteine-rich peptides (insect defensins and drosomycins), proline-rich peptides (apidaecins, drosocins, and lebocins), and glycine-rich peptides/proteins (attacins and gloverins). Defensins, cecropins, proline-rich peptides, and attacins are widespread, while gloverins and moricins have been identified only in Lepidoptera. However, it is worth noting that many other insect AMP families may still be unknown due to their limited taxonomical distribution or lack of research efforts. Most active AMPs are small peptides comprising 20–50 residues, which are generated from larger inactive precursor proteins or pre-proteins, whereas gloverins (ca. 14 kDa) and attacins (ca. 20 kDa) are medium-sized antimicrobial proteins [5].

In Coleoptera, i.e., the most diverse order of insects [15], AMPs have been isolated in many species representing several families, mainly from the suborder Polyphaga: Tenebrionidae [16], Cerambycidae [17] Scarabaeidae [18], Silphidae [19], Chrysomelidae [20], Nitudulidae [21,22], and Curculionidae [23], and isolated recently from the family Cicindelidae, suborder Adephaga [13]. Some of these papers conclude that the isolated oligopeptides show activity mainly against Gram-positive bacteria [24] and, to a lesser extent, against some Gram-negative bacteria [6]. Many beetle species are pests. Currently, biological control based mainly on the use of entomopathogenic bacteria and fungi is the most effective method to fight the pests [13,25,26]. Among beetles, the red palm weevil (RPW) is the most troublesome pest [27].

*Rhynchophorus ferrugineus* (Olivier, 1791, Coleoptera: Curculionidae) is a particularly invasive and destructive palm pest affecting more than 20 different palm species, including date palm, Canary Island date palm, coconut palm, and African oil palm. It mostly attacks young plants at the age of 20 years or younger [28]. During the last two decades, the species has rapidly extended its native geographical range from the Indian sub-continent and southeast Asia into date palm-growing countries of Africa, the Middle East, and the Mediterranean Basin, most likely by multiple accidental anthropogenic introductions [27,28,29,30] or during independent invasion events [31]. Adult females oviposit in wounds, cracks, and crevices on the trunks of date palms. Hatched larvae (grubs) chew the surrounding plant tissue and penetrate into the interior of palm trunks, leaving behind frass (plant fibers), which cause enormous and even fatal damage to the plant. Larval feeding in the trunk of infested palms usually leads to tree death. Following introduction, RPW has become the major pest of date palm and has had a serious impact on the date palm industries, causing large economic losses worldwide for the last 30 years [28,32]. So far, there have been many studies on the management of RPW [33,34,35,36,37,38]. However, finding effective control methods for this vicious pest still requires further research. The antimicrobial molecules of RPW have also been the subject of many different studies. Abdally et al. detected the presence of lectins in the midgut of RPW larvae and adults [39]. Mazza et al. investigated the antimicrobial activity of RPW eggs and the integument of larvae and adults and they found that the polar surface fraction of cuticle extracts inhibited the growth of Gram-positive bacteria and the entomopathogenic fungus *Beauveria bassiana* [40]. Recent studies have also shown that intestinal extracts from RPW larvae inhibited Gram-positive bacteria (*Enterococcus faecalis* and *Staphylococcus aureus*), Gram-negative bacteria (*E. coli* and *Klebsiella* spp.), and fungi (*Candida albicans* and *Penicillium* sp.) [41]. 

One of the most important recent achievements in research on RPW was the study conducted by Wang et al., who performed the first large-scale de novo cDNA library sequencing and annotated the results based on the known insect genes and *Tribolium castaneum* genome assembly [33]. This work is essential for further detailed molecular and proteomic studies on RPW. In the present paper, we isolated and identified AMPs that function in the hemolymph of RPW. Because majority of insect AMPs are produced by the fat body tissue and excreted into the hemolymph in response to infection or injury, the studied AMPs were isolated from hemolymph of beetles immunized previously by injection of a mixture of live *E. coli* and *M. luteus* cells. After isolation of AMPs, verification of their bactericidal activity and identification by N-terminal sequencing or by mass spectrometry, we performed bioinformatic analysis of obtained sequences based on the aforementioned transcriptome database. Obtained results provide new insights into the defense response in Coleoptera, particularly in pests. 

## 2. Materials and Methods

### 2.1. Insect Sampling and Immunization

Adult RPW beetles, without sex indication, were collected in June 2019 from infested Canary Island date palm (*Phoenix canariensis*) fields in El Kassasin District, Ismailia Governorate in Egypt (northeastern part of the country). The insects were reared for a short time in sugar cane-containing plastic boxes in the dark at room temperature. Each beetle was identified as *Rhynchophorus ferrugineus* (Olivier, 1790) (Coleoptera: Curculionidae: Dryophthorinae: Rhynchophorini) with the use of all morphology identification keys [27,29,30]. In total, 79 individuals were immunized by injection into the hemocoel of 2 μL of liquid phosphate buffered saline (PBS) containing a suspension of live *Escherichia coli* K12/ATCC 10,798 and *Micrococcus luteus* ATCC 4698 cells (2 × 10^7^ CFU/mL). As a non-immunized control, 12 individuals were only pierced on the thorax with a sterilized needle. After 24 h of the immune challenge, 5 adults died and another 2 individuals were not analyzed. All individuals in the control group survived. 

### 2.2. Hemolymph Collection

Hemolymph samples were collected into Eppendorf tubes containing an equal volume of PBS with 0.1% (*w*/*v*) phenylthiourea (PTU, melanization inhibitor), mixed, and deprived of cellular components by two subsequent centrifugation steps: at 5000× *g* for 15 min and then at 20,000× *g* for 20 min, both at 4 °C. The clear supernatant was frozen and stored for further analyses.

### 2.3. Chromatographic Separations

**Figure 1:**
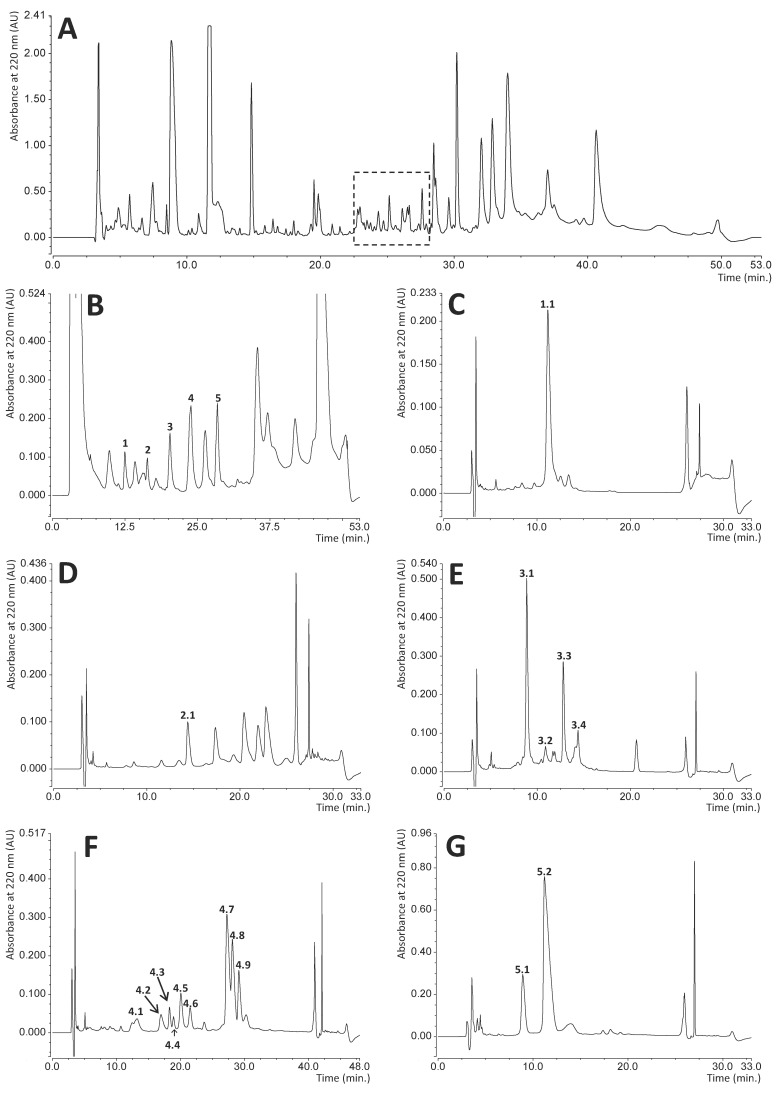
Reversed-phase high pressure liquid chromatography (RP-HPLC) of the components of the hemolymph of immunized insects. Panel (**A**) shows the full elution profile of the hemolymph. All visible peaks form this separation were collected and tested for antibacterial activity against *Escherichia coli* and *Staphylococcus intermedius.* The dashed square indicates the region with the highest activity. Panel (**B**) presents further separation of this specific region into several fractions. Of these fractions, five peaks marked from 1 to 5 had bactericidal activity. Panels (**C**–**G**) show individual separations of these fractions into subfractions containing homogeneous compounds. Those exhibiting bactericidal activity are marked with double digit numbers and were used for further SDS-PAGE analysis and identification. The details of hemolymph collection and chromatography conditions are described in the Materials and Methods section.

All reversed-phase high-pressure liquid chromatography (RP-HPLC) separations were performed using Ultimate 3000 apparatus (Thermo Scientific, Waltham, MA, USA) equipped with a Discovery Bio Wide Pore C18 4.6 × 250 mm column (Sigma, St. Louis, MI, USA). Two solvents were applied: A—0.1% (*v*/*v*) trifluoroacetic acid (TFA) in water and B—0.07% TFA, 80% acetonitrile (both *v*/*v*) in water. The spectrophotometric detection at 220 and 280 nm was carried out at a flow rate of 1 mL/min. The following linear gradient steps were designed:

- Total 0–100% B for 20 min for fast comparative analyses of hemolymph shown in Supplementary Materials Figure S1;
- Total 0–75% B for 40 min for separation of hemolymph shown in Figure 1A;
- Total 30–55% B for 40 min for separation of fractions 1–5 shown in Figure 1B;
- Total 33–35% B for 20 min for separation of subfractions 1.1 and 2.1 shown in Figure 1C,D;
- Total 35–45% B for 20 min for separation of subfractions 3.1–3.4 shown in Figure 1E;
- Total 35–40% B for 35 min for separation of subfractions 4.1–4.9 shown in Figure 1F;
- Total 40–43%B for 20 min for separation of subfractions 5.1 and 5.2 shown in Figure 1G.

Before each separation, the column was equilibrated at the starting percentage of solvent B. After completion of the gradient, the column was regenerated for 5 min at 100% B. During separations, the fractions and subfractions were collected manually into plastic tubes, evaporated in a vacuum centrifuge, and dissolved in water for further analyses.

### 2.4. Antibacterial Radial Diffusion Assay

The bactericidal or bacteriostatic activity of collected fractions was evaluated using the radial diffusion assay. Two subsequent 5 μL portions of relevant solutions were pipetted onto tryptic soy (TSB) plates solidified with 0.75% agarose (low EEO grade, Sigma, St. Louis, MI, USA) containing a 200× diluted overnight culture of *Escherichia coli* K12/ATCC 10,798 or *Staphylococcus intermedius* ATCC 29663. Following overnight incubation at 37 °C, the antibacterial activity was evaluated visually and non-quantitatively: compounds causing clear inhibition zones were presumed as bactericidal, while compounds causing partial clearance were regarded as bacteriostatic ones.

### 2.5. Protein Chemistry Techniques

**Figure 2:**
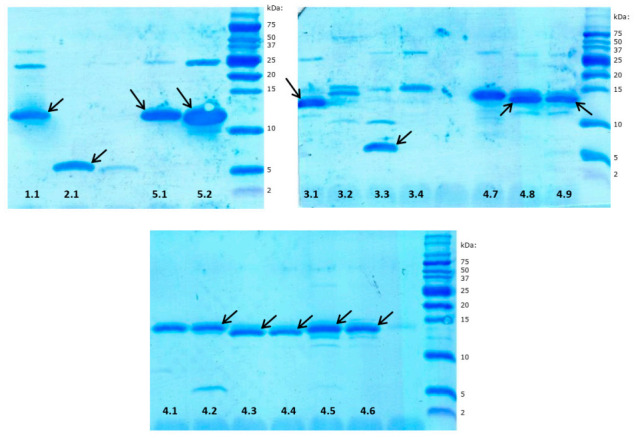
Sodium dodecyl sulphate polyacrylamide gel electrophoresis (SDS-PAGE) image of immune peptides and proteins isolated from *Rhynchophorus ferrugineus* hemolymph. The numbers correspond to the subfractions shown in Figure 1C–G. Selected bands denoted by arrows were identified by N-terminal amino acid sequencing after excision from the polyvinylidene difluoride (PVDF) membrane. The other proteins from subfractions 3.2, 4.1, and 4.7 were identified by mass spectrometry.

Denaturing sodium dodecyl sulphate polyacrylamide gel electrophoresis (SDS-PAGE) was performed in reducing conditions using Tris-Tricine peptide-separating gels [42]. After electrophoresis, the proteins were electrotransferred onto polyvinylidene difluoride (PVDF) membrane (Immobilon PSQ, 0.22 μm pore size, Millipore, Burlington, MA, USA) using 10 mM N-cyclohexyl-3-aminopropanesulfonic acid (CAPS) buffer, pH 11.0, containing 10% (*v*/*v*) methanol, and subsequently stained with Coomassie Blue. The protein bands (denoted by arrows in Figure 2) were excised from the membrane and their N-terminal amino sequences were determined by Edman degradation using a PPSQ-31A (Shimadzu, Kyoto, Japan) automatic protein sequencer.

### 2.6. Mass Spectrometry

Protein identification by mass spectrometry was performed for liquid subfractions 3.2, 4.1, and 4.7 (Figure 1E,F) as described in [43] with minor changes. Briefly: disulfide bridges were reduced by dithiotreitol (5 mM final concentration), and cysteines were subsequently blocked by iodoacetamide (5 mM final concentration). Both reactions were carried out at 60 °C in 20 mM ammonium bicarbonate buffer (pH 7.5) for 10 min each. Afterwards, overnight digestion by trypsin (Promega Gold, 50 fmol/sample) was performed followed by lyophilization and resuspension of the dry residue in chromatography solvent A (2:97.9:0.1 acetonitrile: water: formic acid, *v*/*v*/*v*). NanoLC-MS/MS separations were done on an Ultimate 3000 capillary liquid chromatograph (Thermo Scientific, Waltham, MA, USA) connected on-line to an AmaZon SL mass spectrometer (Bruker, Billerica, MA, USA). The separation conditions were as follows: solvent A as described above, solvent B (50:49.9:0.1 acetonitrile: water: formic acid, *v*/*v*/*v*), and linear gradient steps: 0 min 4% B, 37 min 55% B, 37.5 min 70% B, 38.5 min 70% B, 39 min 4% B, and 40 min 4% B. A 300 µm ID × 5 mm precolumn and a 75 µm ID × 150 mm capillary column, both C18 PepMap 100, 5 µm (Thermo Scientific, Waltham, MA, USA) were used. The mass spectrometer settings were as follows: scan range 600–1800 m/z, ICC Target 300,000 ions, ion source: ESI nano sprayer, capillary voltage 4200 V, nebulizer pressure 10 psi, gas temp. 140 °C, gas flow 4 L/min. Fragmentation settings: precursor ions 2, threshold intensity 300,000, preferred isolation of doubly charged ions, active exclusion after two MS^2^ spectra for 30 s. MS^2^ spectra were acquired in the range of 300–2000 m/z in the maximum resolution mode. The following software was applied for data analysis: Chromeleon Xpress with DCMS Link (Thermo Scientific, Waltham, MA, USA), TrapControl ver. 8.0 (Bruker, Billerica, MA, USA), both supervised and coordinated by Compass HyStar 4.1 SR1 (Bruker, Germany). Data extraction from the raw result files was done using Compass DataAnalysis 4.4 SR1 (Bruker, Billerica, MA, USA). Final *.mgf files usually containing approximately 500 fragmentation spectra were sent to the Mascot Search Engine (Matrix Science Ltd., London, UK). The Mascot MS/MS ion search settings were as follows: database: NCBIprot; taxonomy: all entries; enzyme: trypsin (with 1 missed cleavage allowed); fixed modifications: carbamidomethylation; variable modifications: methionine oxidation; peptide tolerance: +/−1.2 Da; MS/MS tolerance +/−0.6 Da; ^13^C = 1; peptide charge: 1+,2+,3+; instrument: ESI-TRAP.

### 2.7. Bioinformatic Techniques

The cDNA sequences of the *Rhynchophorus ferrugineus* transcriptome were obtained from the National Center for Biotechnology Information (NCBI) Nucleotide database (accession numbers from JR467464 to JR494080). All analyses were carried out in Jupyter Notebook environment release 6.0.1 [44,45]. The sequences were searched for fragments coding for the analyzed peptides using a translated BLAST tool (tblastn, BLAST+, v. 2.9.0) [46] at the default E-value threshold of 10. Using in-house Python scripts utilizing Pandas library [47], the search results were filtered to contain only hits of 100% identity to the query peptide sequences. Each BLAST hit location was extended both downstream to a stop codon (TAA, TAG, TGA) or upstream up to the furthest possible initiation codon (preferentially ATG or TTG/CTG if ATG was not present) to uncover the putative coding sequence (CDS). The products of the coding sequences were searched in CLC Main Workbench (8.1.3, Qiagen) using a plugin for SignalP 4.1 [48] to determine the presence and the extent of putative signal peptides. The identity of CDS products was determined using translated BLAST as described above to search the non-redundant NCBI Nucleotide database obtained from the NCBI FTP server (ftp://ftp.ncbi.nlm.nih.gov/blast/db/). The results were filtered accordingly to select hits with the highest statistical significance, i.e., those with the lowest E-value. The information on CDS locations, cross-referenced accession numbers of the protein/peptide product, and the source organisms was obtained directly from respective NCBI Nucleotide database entries using in-house Python scripts and NCBI E-utilities.

## 3. Results

RP-HPLC chromatography is a convenient technique facilitating effective separation and quantitation of different immune polypeptide factors present in various insect hemolymph extracts [49,50,51,52]. This technique was also applied in this study to identify bactericidal or bacteriostatic peptides and proteins excreted by adult RPW individuals into the hemolymph. During preliminary experiments, we compared the chromatographic profiles of hemolymph samples collected from both control (healthy) insects and infected beetles 24 h after injection of a mixture of Gram-negative and Gram-positive bacteria (*Escherichia coli* and *Micrococcus luteus*) into the hemocoel. The RP-HPLC profiles were determined for hemolymph sampled from four control and six immunized insects, but the chromatograms obtained did not allow unambiguous identification of peaks whose intensity increase could be attributed only to immunization (Supplementary Materials Figure S1). However, the antibacterial radial diffusion assay performed on fractions collected during separation of immunized hemolymph distinguished a group of peaks (dashed square in Figure 1A) that indicated bactericidal or bacteriostatic activity towards the standard bacterial strains used: *E. coli* and *Staphylococcus intermedius*. The compounds eluted in this region were further separated, and the successive antibacterial tests indicated five main fractions with activity towards bacteria (peaks 1–5 shown in Figure 1B). All these peaks were individually collected and fractionated in a series of different individually optimized gradient separations (Figure 1C–G) into homogenous final subfractions 1.1, 2.1, 3.1–3.4, 4.1–4.9, and 5.1–5.2. All these subfractions showed different degrees of bactericidal or bacteriostatic activity towards *E. coli* and/or *Staphylococcus intermedius* strains (evaluated by the qualitative radial diffusion assay, Supplementary Materials Figure S2), while the SDS-PAGE analysis of these compounds demonstrated that they form a group of peptides and relatively small proteins of molecular mass in the range from approx. 4 to 15 kDa (Figure 2). Most of them are homogenous, while some (subfractions 1.1, 5.2, 3.1–3.3, and 4.2) contain approx. 10% of impurities.

Most of these compounds (subfractions 1.1, 2.1, 3.1, 3.3, 4.2–4.6, 4.8, 4.9, 5.1, 5.2, denoted by arrows in Figure 2) were identified by determination of their N-terminal amino acid sequences by direct chemical sequencing using Edman degradation. The analyses were performed using bands excised from the PVDF membrane. In all cases, it was possible to determine the sequence of 15 to 25 residues (Table 1). However, sequencing was impossible in the case of subfractions 3.2, 4.1, and 4.7, proving that these proteins bear a chemical blockade at the alpha-amino group of the N-terminal amino acid. These three compounds were identified with the bottom-up proteomic approach, using mass spectrometry. The results allowed determination of a 12–24 residue-long amino acid sequence of 2 to 5 internal peptides from each subfraction (Table 1). In the case of fraction 3.4, which contained a protein of molecular mass of approx. 15 kDa, both sequencing and mass spectrometry identification attempts failed.

The bioinformatic analyses revealed that all determined amino acid sequences have 100% identity to those encoded in corresponding nucleotide sequences identified several years ago in the first large-scale full-length cDNA sequencing project for *R. ferrugineus* [33] and in different individual nucleotide sequences of particular mRNA obtained independently from *R. ferrugineus*. Furthermore, we also showed significant sequence similarities for corresponding bactericidal peptides and proteins found in other insects. In sum, the identified factors are two peptides similar to defensins, one cecropin-A1-like peptide, one attacin-B-like protein, five isoforms of pheromone-binding proteins, and seven isoforms of odorant-binding proteins. All essential information about the identified proteins and peptides is collected in Table 1, which also includes the determined amino acid sequences, accession numbers of respective cDNA sequences of *R. ferrugineus* transcriptome, theoretical molecular masses of the encoded mature proteins or peptide products, and data on the most similar proteins and peptides found in other insect species or in other studies of *R. ferrugineus*. More detailed information concerning the bioinformatic parameters of the analyzed sequences can be found in Supplementary Materials Table S1.

In the case of subfraction 1.1, the determined 15-mer N-terminal amino acid sequence allowed to identify of three coding sequences (CDS) in the *R. ferrugineus* transcriptome for this particular peptide motif: JR492050.1, JR485705.1, and JR485629.1. These sequences encode three similar proteins of molecular mass of approx. 12 kDa. All of them share approx. 55% of identical residues with attacin-B-like protein from *Dendroctonus ponderosae*.

In the case of subfractions 2.1 and 3.3, the determined 19-mer N-terminal amino acid sequences allowed to identify of three *R. ferrugineus* transcriptome cDNA sequences: JR471060.1, JR491618.1, and JR477230.1. They encode three 63-, 65-, and 44-amino acid-long peptides, respectively, of molecular mass in the range of 6.20–7.25 kDa. They have 56 to 85% of identical residues (69–92% similarity) to defensin from *Sitophilus zeamais*.

The 19-mer N-terminal amino acid sequence of the peptide from subfraction 4.2 was identical to that encoded in nucleotide sequence JR486084.1 found in the *R. ferrugineus* transcriptome, which is a 4.97 kDa and 44-mer-long peptide demonstrating 73.91% of sequence identity to a hypothetical antimicrobial peptide from *Sitophilus zeamais*. This peptide also shares some degree of similarity with the predicted sequence of cecropin-A1-like peptide of *Dendroctonus ponderosae*.

For subfractions 3.1, 4.3, 4.4, 4.8, and 4.9, we were able to determine 20–25 amino acid-long N-terminal sequences which all are identical to motifs present in two 13,187 and 12,840 Da proteins encoded in two respective cDNA sequences, namely sequence JR470869.1 and JR489305.1, from the *R. ferrugineus* transcriptome. The protein product of the JR470869.1 sequence, i.e., a protein from subfraction 3.1, has over 74% of identical residues with pheromone-binding protein 14 from *Cyrtotrachelus buqueti*. In turn, the product of the JR489305.1 gene (corresponding to proteins from subfractions 4.3, 4.4, 4.8, and 4.9) has over 79% identity with pheromone-binding protein 10, also from *C. buqueti*. Moreover, the presence of four different protein subfractions with the same N-terminal sequences encoded by the same gene indicates that subfractions 4.3, 4.4, 4.8, and 4.9 contain isoforms of the same protein trimmed proteolytically at the C-terminus or bearing different posttranslational modifications.

In the case of subfractions 3.2, 4.1, 4.5, 4.6, 4.7, 5.1, and 5.2, we identified N-terminal or internal amino acid sequences identical to those encoded by three different CDS from the *R. ferrugineus* transcriptome: JR484067.1, JR472381.1, and JR482588.1. These sequences were also independently identified in several *R. ferrugineus* mRNA isolations and deposited under accession numbers KY653085.1, KT748815.1, and KY653084.1, respectively. The protein products coded by these CDS were described as odorant-binding proteins 9, odorant-binding protein, and odorant-binding protein 28, respectively.

## 4. Discussion

The well-known insect bactericidal factors identified in our study were represented by one attacin-B-like protein, two peptides similar to defensins, and one cecropin-A1-like peptide. The attacin-B-like protein was found in subfraction 1.1. Attacins are glycine-rich, bacteriostatic, medium-size (ca. 20 kDa) proteins which interact with lipopolysaccharides; hence, their action is directed mainly at defense against Gram-negative bacteria, with the greatest effectiveness against *Escherichia coli* [5,53,54]. These proteins have a negligible hemolytic effect on red blood cells; therefore, they are considered as potential alternatives to antibiotics [5,55,56]. They are synthesized as pre-pro-peptides with an N-terminal signal sequence, a P domain, an attacin domain, and two glycine-rich (G1 and G2) domains at the C-terminus. Attacins were first discovered in the Lepidoptera [57,58], but they are also common in other insect orders [5]. We were able to detect a single attacin-like protein in the immunized hemolymph of RPW; however, in the current version of the *R. ferrugineus* transcriptome, three different CDS code for products that contain the motif found in the N-terminal sequence of the isolated protein. These sequences encode three proteins of the same molecular mass of approx. 12 kDa. Most probably, only one of them is expressed and detected in the hemolymph of RPW. All these proteins share relatively small sequence similarity to the gene of the predicted attacin-B-like protein (unpublished, NCBI reference sequence No XP_019761640.1) identified in mountain pine beetle *Dendroctonus ponderosae* during whole genome shotgun sequencing. However, in the case of the putative RPW attacin found in subfraction 1.1, the low sequence similarity to other known attacins (only 55% of identical residues) necessitates further research on the function and role of this protein in the *R. ferrugineus* immune response.

Defensins are a family of small cationic peptides with potent antimicrobial activity and a molecular mass of about 4 kDa (approx. 40 amino acids in length), usually containing six cysteines forming three disulfide bridges [59]. They are found in various living organisms, including humans, other mammals, birds, reptiles, fish, mollusks, arthropods, many different groups of insects, plants, and fungi. The structures and mechanisms of the antimicrobial action of defensins have been broadly described, and possible applications of these peptides have been comprehensively discussed by many authors [60,61,62,63]. However, the structure of the insect defensin molecule is quite characteristic, as it usually consists of an N-terminal loop, an α-helix, and anti-parallel β-sheets at the C-terminus, where two disulfide bridges connect the helix with the first β-sheet and the third bridge connects the loop with the second β-sheet. Defensins are most active against Gram-positive bacteria, including such human pathogens as *Staphylococcus aureus* [5,64,65]. As demonstrated earlier, we discovered three isoforms of these peptides (63-, 65-, and 44-amino acid-long) in the RPW hemolymph, with pronounced similarities to defensin in grain weevil *Sitophilus zeamais*. The weevil defensin has not been characterized at the protein level to date, but its genes were identified by Anselme et al. in a study of the immune response of the weevil to mutualistic endosymbiotic intracellular bacteria [23]. The authors of this study hypothesize that the weevil defensin limits infections by endosymbionts only to the specialized bacteria-bearing tissue of the insect host.

Cecropins are alpha-helical linear peptides without cysteines, containing 31–42 amino acid residues and having individual names, depending on the taxa in which they were detected. They were discovered for the first time in the Lepidoptera and have been described in other insect orders as well, including Coleoptera and Diptera. Cecropins exhibit a broad spectrum of antimicrobial activity against both Gram-positive and Gram-negative bacteria as well as fungi [63]. Cecropins also exhibit a number of other properties, e.g., immunomodulatory [66,67] and toxic activity against tumor cells [67]. The RPW peptide found in subfraction 4.2 has a similar sequence to the hypothetical antimicrobial peptide of *Sitophilus zeamais* identified during the aforementioned studies of weevil tolerance of endosymbionts [23]. However, the sequence of this hypothetical peptide is highly similar to numerous cecropins from different insects. The sequence of peptide 4.2 is also similar to the sequence of putative cecropin-like protein A1 found in the genome of mountain pine beetle *Dendroctonus ponderosae* (unpublished, NCBI reference sequence No XP_019757573.1).

Interestingly, in the immunized RPW hemolymph, we unexpectedly identified some other bactericidal factors as well. These were five isoforms of pheromone-binding proteins and seven isoforms of odorant-binding proteins. The term “odorant-binding proteins” (OBPs) usually describes proteins that are unique in terms of their number, abundance, and diversity in the olfactory system of various insects and which are able to bind odorous substances [68]. The first insect OBP was found as a sex pheromone-binding protein in an antennal extract of the giant silk moth *Antheraea polyphemus* [69,70]. Soon, mammalian OBPs were discovered. They have similar function but a completely different structure than insect OBPs [71,72]. The vertebrate proteins belong to the lipocalin superfamily and therefore represent a structurally different class to the OBPs of Hexapoda [73,74]. Within the Hexapoda, the number of genes encoding OBPs is highly variable among species [75]. For example, within the Entognatha, a larger number of genes coding for OBPs have been reported for Collembola in comparison to Protura and Diplura [76].

The odorant-binding proteins are relatively small (10 to 30 kDa in size), water-soluble, and uniquely expressed in the olfactory tissue of insects and vertebrates [72,77]. They can be grouped into general odorant-binding proteins, pheromone-binding proteins, and antennal binding protein X [69,78]. The insect OBPs contain mainly α-helical domains, which define the hydrophobic binding cavity and are divergent across and within species [74,79,80]. The structure of OBP molecules is stabilized by three disulfide bridges between the conserved pattern of six cysteines [81,82,83]. The family of OBPs includes members with a smaller (C-minus OBPs) or higher number (C-plus OBPs) of cysteines and atypical OBPs containing additional domains [84,85]. Alongside other proteins, such as chemosensory proteins, odorant receptors, ionotropic receptors, sensory neuron membrane proteins, and odorant degrading enzymes, OBPs belong to the main proteins of the peripheral olfactory system in insects [78,86]. This family has been found mainly in Lepidoptera (butterflies and moths) but also in other insect orders, and the genomic analysis of *Drosophila* and other insect species, e.g., *Anopheles gambiae, Apis mellifera, Bombyx mori*, and *Tribolium castaneum*, has revealed that the OBP genes significantly differ between species [87,88,89].

The odorant-binding proteins are mostly and abundantly expressed in the antennae [90,91,92], including the taste system and chemosensory organs [93,94,95]. They are also present in reproductive organs [96] and are produced in the sperm and transferred to females during mating [97,98,99,100]. They are also known to be ectopically expressed in such tissues as the gut [101]. Functionally, the insect OBPs are involved in the detection of both general odorants and sex pheromones; they capture and transport them to receptor neurons [70,89,102,103]. Some OBPs are hypothesized to hasten odor response termination by extracting odorant molecules from the sensillar lymph or from receptors themselves [104]. It has also been hypothesized that OBPs are part of the molecular coding of odors and pheromones by forming specific complexes with odorant molecules that could ultimately stimulate olfactory receptors to trigger the olfactory transduction cascade [105]. However, an increasing body of evidence reveals a much broader role for this family of proteins [68,78,106], and OBPs are thought to have multiple roles, besides olfaction, in reproduction, egg laying, and anti-inflammatory responses [96]. Quite recently, Bianchi et al. have shown that vertebrate OBPs exhibit antimicrobial activity against *Candida albicans, Pseudomonas aeruginosa*, and several other bacterial and yeast strains and suggested that this activity may be related to scavenging several compounds important for bacteria, such as quorum-sensing peptide-pheromones, N-acyl-homoserine lactones, furanones, hormones, quinolones, and fatty acids [107]. The authors of the aforementioned study hypothesized about the role of OBPs in the anti-infective immunity of vertebrates, because OBPs are synthesized in all tracts of the respiratory apparatus and are secreted at millimolar levels into the mucus layer of the epithelium. In our study, we have identified for the first time OBPs in the immunized hemolymph of the adult *Rhynchophorus ferrugineuss*. This finding confirms that also insect OBPs have antibacterial activities and that their level increases in the hemolymph after immunization. However, the detailed role of OBPs in anti-infective insect immunity and their mechanism of action toward pathogenic bacteria need further separate and detailed verification.

The second surprising group of new antibacterial factors found in this study in the immunized RPW hemolymph is the pheromone-binding proteins. We have found these proteins in as many as seven isolated subfractions. They are most probably proteolytically trimmed protein products of three different *R. ferrugineus* CDS. The pheromone-binding proteins are a subtype of odorant-binding proteins mediating the early stages of detection of volatiles in both insects and vertebrates, with the major function of pheromone binding [69,75,86,108,109,110]. They represent a family of proteins related to insect sex pheromone recognition identified in many species representing different insect orders [110,111,112]. They are small (14–20 kDa), water-soluble, extracellular proteins of around 130–150 amino acids, containing six or seven alpha helices that form a conical binding cavity and six cysteine residues that form three disulfide bonds stabilizing the three-dimensional structure [83,113]. The pheromone-binding proteins are located in the male antennal long olfactory trichoid sensilla among several proteins involved in insect olfactory recognition [86,91]; however, lower levels of expression of these proteins have also been found in female antennae [110]. Besides the antennae, pheromone-binding proteins were also identified in other appendages, e.g., the proboscis, labial palps, and legs [114,115] and in the sex pheromone gland of some Lepidoptera moths [116,117]. The pheromone-binding proteins are synthesized by two olfactory accessory cells: trichogen and tormogen cells and are secreted abundantly into the sensillum lymph of trichoid sensilla [118,119,120].

Studies on insect pheromone-binding proteins indicate that they are multifunctional: they act as solubilizers and carriers of hydrophobic pheromones in the aqueous sensillum lymph, concentrate odorants in the sensillum lymph, protect pheromones from enzymatic degradation, and serve as cofactors in the activation of pheromone receptors. Furthermore, they are involved in the postulated odorant molecule deactivation and thereby in facilitation of their transport to the receptor neurons, enhancing the sensitivity of olfactory receptors to sex pheromones [106,121]. Hence, pheromone-binding proteins play important roles in the information exchange between insect sexes, specifically in the process of transporting fat-soluble odor molecules from the external environment to olfactory receptors through the olfactory sensillum lymph. The functions of pheromone-binding proteins in this process may explain the sex pheromone identification mechanism used by insects, laying a theoretical foundation for the prevention and control of pests by interfering with olfactory recognition. A study conducted by McKenna et al. [91] suggests the possibility that pheromone-binding proteins are members of a larger class of proteins, extending beyond the olfactory system. In weevils, pheromone-binding proteins have been well studied in *Cyrtotrachelus buqueti* [112,122,123]. In this beetle, the pheromone-binding protein has dual roles in the processes of sensing sex pheromones and plant volatiles [123]. The phylogenetic analysis showed that *C. buqueti* pheromone-binding proteins are similar to pheromone-binding proteins of other insects, for example, the similarity to pheromone-binding proteins from Coleoptera reaches 38.47% [112]. On the other hand, the identified amino acid sequences of the bactericidal proteins from immunized hemolymph of *Rhynchophorus ferrugineus* show a high degree of similarity to the *C. buqueti* pheromone-binding proteins. In fact, these weevil species are closely related phylogenetically, representing the same subtribe Rhynchophorina [124].

In *Rhynchophorus ferrugineus*, which is a widely distributed and highly destructive pest of palms, the pheromone-binding proteins play an especially important role in the process of olfactory recognition of plants. In this study, we have also proved that these proteins have antibacterial properties and their level increases in the hemolymph after immunization. To the best of our knowledge, this is the first report of the bactericidal properties of this class of proteins and, as in the case of OBPs, this phenomenon needs separate detailed verification, especially in terms of the mechanism of bactericidal activity and the role in anti-infective response. Since pheromone-binding proteins and OPBs are closely functionally and phylogenetically related, one may expect that their role in insect immunity is similar. Moreover, the fact that both OBPs and pheromone-binding proteins were found in the immunized hemolymph, beyond the typical tissues in which they are expressed, suggests that these proteins may have acquired new functions along evolution. One can also speculate that they have adopted an alternative gene expression control system in comparison with regular OBPs or pheromone-binding proteins. Since pheromone-binding proteins are strongly male-specific [110], both their level and the role in the hemolymph should also be verified in the context of the sex of the insects as well as the sexual maturity status.

## 5. Conclusions

The present work is the first comprehensive study focused on identification of bactericidal proteinaceous factors produced by red palm weevil *Rhynchophorus ferrugineus*—a troublesome pest of palms. In immunized hemolymph of this beetle, we found both well-known families of insect bactericidal peptides and proteins, such as attacins, defensins and cecropins, as well as two groups of proteins, which were earlier not known in Insecta to serve functions related to anti-infective response: odorant- and pheromone-binding proteins. Indeed, some recent studies suggest that vertebrate odorant-binding proteins are able to exhibit antimicrobial activity by scavenging selected low-molecular compounds important for bacteria. On the other hand, at present, nothing is known about the mechanism of the bactericidal action of pheromone-binding proteins. However, both odorant- and pheromone-binding proteins have similar functions and phylogeny; therefore, one may expect that their role in insect immunity is similar. The present work provides a base for further detailed studies of the particular role of both protein families in insect response to bacterial infections.

## Figures and Tables

**Table 1:** Amino acid sequences of bactericidal proteins and peptides isolated from *Rhynchophorus ferrugineus* immunized hemolymph and essential bioinformatic parameters of their putative coding sequences.

Fraction	Identified Amino Acid Sequence(s)	Technique of Identification(N-terminal Sequencing MS—Mass Spectrometry)	Database Entry for Nucleotide Sequence in *Rhynchophorus ferrugineus* Transcriptome and Theoretical Molecular Masses of the Mature Protein/Peptide Containing the Determined Peptide Sequence		Sequences in the Non-redundant NCBI Nucleotide Database Coding for the Most Similar Product to the Analyzed Coding Sequence from the *Rhynchophorus ferrugineus* Transcriptome				
					Accession Number	Product Accession Number	Percent of Identical Residues	Product Name	Source Organism
1.1	1.ETKQLNWQPK DDNQP	N-terminal	JR492050.1	11.95 kDa	XM_019906081.1	XP_019761640.1	55.41	Attacin-B-like protein	*Dendroctonus ponderosae*
			JR485705.1 *	12.58 kDa	XM_019906081.1	XP_019761640.1	55.41	Attacin-B-like protein	*Dendroctonus ponderosae*
			JR485629.1 *	12.27 kDa	XM_019906081.1	XP_019761640.1	55.41	Attacin-B-like protein	*Dendroctonus ponderosae*
2.1	1.ATXDLLSFEV KGFKLNDSA	N-terminal	JR471060.1	6.90 kDa	EU282115.1	ABZ80665.1	77.11	Defensin	*Sitophilus zeamais*
3.1	1.LTIEESKEKF KKAHEKXNAD VSTKL	N-terminal	JR470869.1	13.17 kDa	KX814434.1	APG79375.1	74.44	Pheromone-binding protein 14	*Cyrtotrachelus buqueti*
3.2	1.DTPEQTSIDL DACLRK 2.HMLCMMQGIG AVTSDGHISQ DGVK	MS	JR484067.1	14.09 kDa	KY653085.1	ATU47279.1	100.00	Odorant-binding protein 9	*Rhynchophorus ferrugineus*
3.3	1.ATXDLLSFEA FGIKLNDSA	N-terminal	JR491618.1	7.25 kDa	EU282115.1	ABZ80665.1	55.95	Defensin	*Sitophilus zeamais*
			JR477230.1 *	6.20 kDa	EU282115.1	ABZ80665.1	85.42	Defensin	*Sitophilus zeamais*
3.4	Impossible for identification	-	-	-	-	-		-	-
4.1	1.VSHILKDCAV AK 2.HVVSDESKVS HILK 3.DTPEQTSIDL DACLRK 4.DCAVAKDTPE QTSIDLDACL R 5.HMLCMMQGIG AVTSDGHISQ DGVK	MS	JR484067.1	14.09 kDa	KY653085.1	ATU47279.1	100.00	Odorant-binding protein 9	*Rhynchophorus ferrugineus*
4.2	1.GWLKKQLKSV EKGVRRVRD	N-terminal	JR486084.1	4.97 kDa	EU282118.1	ABZ80668.1	73.91	Hypothetical antimicrobial peptide	*Sitophilus zeamais*
4.3	1.DHVQVRYDNV HKNXQKDPAL YVDDA	N-terminal	JR489305.1	12.84 kDa	KX814430.1	APG79371.1	79.26	Pheromone-binding protein 10	*Cyrtotrachelus buqueti*
4.4	1.DHVQVRYDNV HKNXQKDPAL	N-terminal	JR489305.1	12.84 kDa	KX814430.1	APG79371.1	79.26	Pheromone-binding protein 10	*Cyrtotrachelus buqueti*
4.5	1.LEPNAAAARE SQEKLKQAHQ	N-terminal	JR472381.1	13.84 kDa	KT748815.1	AMK48596.1	100.00	Odorant-binding protein	*Rhynchophorus ferrugineus*
4.6	1.LEPNAAAARE SQEKLKQAHQ	N-terminal	JR472381.1	13.84 kDa	KT748815.1	AMK48596.1	100.00	Odorant-binding protein	*Rhynchophorus ferrugineus*
4.7	1.DTPEQTSIDL DACLRK 2.DCAVAKDTPE QTSIDLDACL R 3.HMLCMMQGIG AVTSDGHISQ DGVK 4.IDEEVFQKLD QNEPVDLPPN FGK	MS	JR484067.1	14.09 kDa	KY653085.1	ATU47279.1	100.00	Odorant-binding protein 9	*Rhynchophorus ferrugineus*
4.8	1.DHVQVRYDNV HKNXQKDPAL YVDDA	N-terminal	JR489305.1	12.84 kDa	KX814430.1	APG79371.1	79.26	Pheromone-binding protein 10	*Cyrtotrachelus buqueti*
4.9	1.DHVQVRYDNV HKNXQKDPAL YV	N-terminal	JR489305.1	12.84 kDa	KX814430.1	APG79371.1	79.26	Pheromone-binding protein 10	*Cyrtotrachelus buqueti*
5.1	1.ATTKSSWNSV HQAXQAKPGV FVDD	N-terminal	JR482588.1	12.41 kDa	KY653084.1	ATU47278.1	100.00	Odorant-binding protein 28	*Rhynchophorus ferrugineus*
5.2	1.ATTKSSWNSV HQAXQAKPGV FVDDA	N-terminal	JR482588.1	12.41 kDa	KY653084.1	ATU47278.1	100.00	Odorant-binding protein 28	*Rhynchophorus ferrugineus*

* Coding sequences that are likely trimmed due to assembly incompleteness or sequencing errors.

## Data Availability

Data is contained within the article and Supplementary Materials.

## Supplementary Materials

The following are available online at https://www.mdpi.com/2218-273X/11/1/83/s1, Figure S1: Comparison of RP-HPLC elution profiles of four control and six immunized cell-free hemolymph samples, Figure S2: Bactericidal or bacteriostatic activities of peptides and proteins isolated from Rhynchophorus ferrugineus hemolymph, Table S1: Detailed information concerning the bioinformatic parameters of bactericidal proteins and peptides isolated from Rhynchophorus ferrugineus immunized hemolymph.

## References

[1] Cantón R., Akóva M., Carmeli Y., Giske C., Glupczynski Y., Gniadkowski M., Livermore D., Miriagou V., Naas T., Rossolini G. (2012). Rapid evolution and spread of carbapenemases among Enterobacteriaceae in Europe. Clin. Microbiol. Infect..

[2] Falagas M.E., Lourida P., Poulikakos P., Rafailidis P.I., Tansarli G.S. (2013). Antibiotic Treatment of Infections Due to Carbapenem-Resistant Enterobacteriaceae: Systematic Evaluation of the Available Evidence. Antimicrob. Agents Chemother..

[3] Babu P., Pulicherla K., Rekha V., Nelson R., Rao K. (2008). Studies on Designing, Construction, Cloning and Expression of a novel synthetic antimicrobial peptide. Curr. Trends Biotechnol. Pharm..

[4] Barbault F., Landon C., Guenneugues M., Meyer J.-P., Schott V., Dimarcq J.-L., Vovelle F. (2003). Solution Structure of Alo-3: A New Knottin-Type Antifungal Peptide from the Insect *Acrocinus longimanus*. Biochemistry.

[5] Yi H.-Y., Chowdhury M., Huang Y.-D., Yu X.-Q. (2014). Insect antimicrobial peptides and their applications. Appl. Microbiol. Biotechnol..

[6] Wu Q., Patocka J., Kuca K. (2018). Insect Antimicrobial Peptides, a Mini Review. Toxins.

[7] Hilchie A.L., Wuerth K., Hancock R.E.W. (2013). Immune modulation by multifaceted cationic host defense (antimicrobial) peptides. Nat. Chem. Biol..

[8] Schmitt P., Rosa R.D., Destoumieux-Garzón D. (2016). An intimate link between antimicrobial peptide sequence diversity and binding to essential components of bacterial membranes. Biochim. Biophys. Acta (BBA) Biomembr..

[9] Idris A.L., Fan X., Muhammad M.H., Guo Y., Guan X., Huang T. (2020). Ecologically controlling insect and mite pests of tea plants with microbial pesticides: A review. Arch. Microbiol..

[10] Lommen S.T., De Jong P.W., Pannebakker B.A. (2016). It is time to bridge the gap between exploring and exploiting: Prospects for utilizing intraspecific genetic variation to optimize arthropods for augmentative pest control—A review. Èntomol. Exp. Appl..

[11] Riegler M. (2018). Insect threats to food security. Science.

[12] Wingfield M., Brockerhoff E.G., Wingfield B.D., Slippers B. (2015). Planted forest health: The need for a global strategy. Science.

[13] Rodríguez-García M.J., García-Reina A., Machado V., Galián J. (2016). Identification, structural characterisation and expression analysis of a defensin gene from the tiger beetle *Calomera littoralis* (Coleoptera: Cicindelidae). Gene.

[14] Bulet P. (2005). Insect Antimicrobial Peptides: Structures, Properties and Gene Regulation. Protein Pept. Lett..

[15] Bouchard P., Bousquet Y., Davies A.E., Alonso-Zarazaga M.A., Lawrence J.F., Lyal C.H.C., Newton A.F., Reid C.A.M., Schmitt M., Ślipiński S.A. (2011). Family-Group Names In Coleoptera (Insecta). ZooKeys.

[16] Welchman D.P., Aksoy S., Jiggins F., Lemaitre B., Jiggins F.M. (2009). Insect Immunity: From Pattern Recognition to Symbiont-Mediated Host Defense. Cell Host Microbe.

[17] Ueda K., Imamura M., Saito A., Sato R. (2005). Purification and cDNA cloning of an insect defensin from larvae of the longicorn beetle, *Acalolepta luxuriosa*. Appl. Èntomol. Zoöl..

[18] Hwang J.-S., Lee J., Kim Y.-J., Bang H.-S., Yun E.-Y., Kim S.-R., Suh H.-J., Kang B.-R., Nam S.-H., Jeon J.-P. (2009). Isolation and Characterization of a Defensin-Like Peptide (Coprisin) from the Dung Beetle, Copris tripartitus. Int. J. Pept..

[19] Hall C.L., Wadsworth N.K., Howard D.R., Jennings E.M., Farrell L.D., Magnuson T.S., Smith R.L. (2011). Inhibition of Microorganisms on a Carrion Breeding Resource: The Antimicrobial Peptide Activity of Burying Beetle (Coleoptera: Silphidae) Oral and Anal Secretions. Environ. Èntomol..

[20] Tang B., Chen J., Tang B.-Z., Meng E. (2014). Transcriptome Immune Analysis of the Invasive Beetle *Octodonta nipae* (Maulik) (Coleoptera: Chrysomelidae) Parasitized by Tetrastichus brontispae Ferrière (Hymenoptera: Eulophidae). PLoS ONE.

[21] Vogel H., Badapanda C., Knorr E., Vilcinskas A. (2013). RNA-sequencing analysis reveals abundant developmental stage-specific and immunity-related genes in the pollen beetleMeligethes aeneus. Insect Mol. Biol..

[22] Vilcinskas A., Mukherjee K., Vogel H. (2013). Expansion of the antimicrobial peptide repertoire in the invasive ladybird *Harmonia axyridis*. Proc. R. Soc. B Biol. Sci..

[23] Anselme C., Pérez-Brocal V., Vallier A., Vincent-Monégat C., Charif D., Latorre A., Moya A., Heddi A. (2008). Identification of the Weevil immune genes and their expression in the bacteriome tissue. BMC Biol..

[24] Tonk M., Knorr E., Cabezas-Cruz A., Valdés J.J., Kollewe C., Vilcinskas A. (2015). *Tribolium castaneum* defensins are primarily active against Gram-positive bacteria. J. Invertebr. Pathol..

[25] Wraight S., Ramos M. (2015). Delayed efficacy of *Beauveria bassiana* foliar spray applications against Colorado potato beetle: Impacts of number and timing of applications on larval and next-generation adult populations. Biol. Control..

[26] Yaroslavtseva O., Dubovskiy I.M., Khodyrev V.P., Duisembekov B.A., Kryukov V.Y., Glupov V.V. (2017). Immunological mechanisms of synergy between fungus *Metarhizium robertsii* and bacteria *Bacillus thuringiensis* ssp. morrisoni on Colorado potato beetle larvae. J. Insect Physiol..

[27] Mahmud A.I., Farminhao J., Viez E.R. (2015). Red Palm Weevil (*Rhynchophorus ferrugineus* Olivier, 1790): Threat of Palms. J. Biol. Sci..

[28] Faleiro J.R. (2006). A review of the issues and management of the red palm weevil *Rhynchophorus ferrugineus* (Coleoptera: Rhynchophoridae) in coconut and date palm during the last one hundred years. Int. J. Trop. Insect Sci..

[29] Giblindavis R.M., Faleiro J.R., Jacas J.A., Peña J.E., Vidyasagar P.S.P.V., Peña J.E. (2013). Biology and management of the red palm weevil, *Rhynchophorus ferrugineus*. Potential Invasive Pests of Agricultural Crops.

[30] Rugman-Jones P.F., Hoddle C.D., Hoddle M.S., Stouthamer R. (2013). The Lesser of Two Weevils: Molecular-Genetics of Pest Palm Weevil Populations Confirm *Rhynchophorus vulneratus* (Panzer 1798) as a Valid Species Distinct from R. ferrugineus (Olivier 1790), and Reveal the Global Extent of Both. PLoS ONE.

[31] Rugman-Jones P.F., Kharrat S., Hoddle M.S., Stouthamer R. (2017). The Invasion of Tunisia by *Rhynchophorus ferrugineus* (Coleoptera: Curculionidae): Crossing an Ocean or Crossing a Sea?. Fla. Èntomol..

[32] Ferry M., Gomez S. (2002). The red palm weevil in the Mediterranean area. Palms.

[33] Wang L., Zhang X.-W., Pan L.-L., Liu W.-F., Wang D.-P., Zhang G.-Y., Yin Y.-X., Yin A., Jia S.-G., Yu X.-G. (2013). A large-scale gene discovery for the red palm weevil *Rhynchophorus ferrugineus* (Coleoptera: Curculionidae). Insect Sci..

[34] Murphy S., Briscoe B. (1999). The red palm weevil as an alien invasive: Biology and the prospects for biological control as a component of IPM. Biocontrol News Inf..

[35] Aldosary N., AlDobai S., Faleiro J. (2016). Review on the Management of Red Palm Weevil *Rhynchophorus ferrugineus* Olivier in Date Palm *Phoenix dactylifera* L.. Emir. J. Food Agric..

[36] Dembilio Ó., Quesada-Moraga E., Santiago-Álvarez C., Jacas J.A. (2010). Potential of an indigenous strain of the entomopathogenic fungus *Beauveria bassiana* as a biological control agent against the Red Palm Weevil, *Rhynchophorus ferrugineus*. J. Invertebr. Pathol..

[37] Mazza G., Francardi V., Simoni S., Benvenuti C., Cervo R., Faleiro J.R., Llácer E., Longo S., Nannelli R., Tarasco E. (2014). An overview on the natural enemies of *Rhynchophorus palm* weevils, with focus on *R. ferrugineus*. Biol. Control..

[38] El Kichaoui A.Y., Abu Asaker B.A., El-Hindi M.W. (2017). Isolation, Molecular Identification and under Lab Evaluation of the Entomopathogenic Fungi *M. anisopliae* and *B. bassiana* against the Red Palm Weevil *R. ferrugineus* in Gaza Strip. Adv. Microbiol..

[39] Abdally M.H., Abo-Elsaad M.M., Al-Shaggag A.A., Al-Bagshy M.M., Al-Shawaf A.A. (2010). Detection of Insect Immunity Substances (Lectins) in the Midgut Extracts from Larvae and Adult Red Palm Weevil *Rhynchophorus ferrugineus* (Olivier) in Al-Ahsa, Saudi Arabia. Pak. J. Biol. Sci..

[40] Mazza G., Arizza V., Baracchi D., Barzanti G.P., Benvenuti C., Francardi V., Frandi A., Gherardi F., Longo S., Manachini B. (2011). Antimicrobial activity of the Red Palm Weevil *Rhynchophorus ferrugineus*. Bull. Insectology.

[41] Sewify G.H., Hamada H., Alhadrami H.A. (2017). In Vitro Evaluation of Antimicrobial Activity of Alimentary Canal Extracts from the Red Palm Weevil, *Rhynchophorus ferrugineus* Olivier Larvae. BioMed Res. Int..

[42] Schägger H., Von Jagow G. (1987). Tricine-sodium dodecyl sulfate-polyacrylamide gel electrophoresis for the separation of proteins in the range from 1 to 100 kDa. Anal. Biochem..

[43] Bodzon-Kulakowska A., Suder P., Mak P., Bierczynska-Krzysik A., Lubec G., Walczak B., Kotlińska J., Silberring J. (2009). Proteomic analysis of striatal neuronal cell cultures after morphine administration. J. Sep. Sci..

[44] Perez F., Granger B.E. (2007). IPython: A System for Interactive Scientific Computing. Comput. Sci. Eng..

[45] Kluyver T., Ragan-Kelley B., Pérez F., Granger B.E., Bussonnier M., Frederic J., Kelley K., Hamrick J., Grout J., Corlay S. (2016). Jupyter Notebooks—A publishing format for reproducible computational workflows. Positioning and Power in Academic Publishing: Players, Agents and Agendas.

[46] Camacho C.E., Coulouris G., Avagyan V., Ma N., Papadopoulos J.S., Bealer K., Madden T.L. (2009). BLAST+: Architecture and applications. BMC Bioinform..

[47] McKinney W. Data Structures for Statistical Computing in Python. Proceedings of the 9th Python in Science Conference.

[48] Nielsen H. (2017). Predicting Secretory Proteins with SignalP. Protein Function Prediction.

[49] Cytryńska M., Mak P., Zdybicka-Barabas A., Suder P., Jakubowicz T. (2007). Purification and characterization of eight peptides from *Galleria mellonella* immune hemolymph. Peptides.

[50] Mak P., Zdybicka-Barabas A., Cytryńska M. (2010). A different repertoire of *Galleria mellonella* antimicrobial peptides in larvae challenged with bacteria and fungi. Dev. Comp. Immunol..

[51] Stączek S., Zdybicka-Barabas A., Mak P., Sowa-Jasiłek A., Kedracka-Krok S., Jankowska U., Suder P., Wydrych J., Grygorczuk K., Jakubowicz T. (2018). Studies on localization and protein ligands of *Galleria mellonella* apolipophorin III during immune response against different pathogens. J. Insect Physiol..

[52] Vertyporokh L., Kordaczuk J., Mak P., Hułas-Stasiak M., Wojda I. (2019). Host-pathogen interactions upon the first and subsequent infection of *Galleria mellonella* with *Candida albicans*. J. Insect Physiol..

[53] Imler J.-L., Bulet P. (2005). Antimicrobial Peptides in Drosophila: Structures, Activities and Gene Regulation. Chem. Immunol. Allergy.

[54] Wei D., Tian C.-B., Liu S.-H., Wang T., Smagghe G., Jia F.-X., Dou W., Wang J. (2016). Transcriptome analysis to identify genes for peptides and proteins involved in immunity and reproduction from male accessory glands and ejaculatory duct of *Bactrocera dorsalis*. Peptides.

[55] Carlsson A., Nyström T., De Cock H., Bennich H. (1998). Attacin—An insect immune protein—Binds LPS and triggers the specific inhibition of bacterial outer-membrane protein synthesis. Microbiology.

[56] Wang L.-N., Yu B., Han G.-Q., Chen D.-W. (2010). Molecular cloning, expression in Escherichia coli of Attacin A gene from Drosophila and detection of biological activity. Mol. Biol. Rep..

[57] Hultmark D., Engström A., Andersson K., Steiner H., Bennich H., Boman H. (1983). Insect immunity. Attacins, a family of antibacterial proteins from *Hyalophora cecropia*. EMBO J..

[58] Lee J., Edlund T., Ny T., Faye I., Boman H. (1983). Insect immunity. Isolation of cDNA clones corresponding to attacins and immune protein P4 from *Hyalophora cecropia*. EMBO J..

[59] Lehrer R.I., Bevins C.L., Ganz T. (2005). Defensins and Other Antimicrobial Peptides and Proteins. Mucosal Immunology.

[60] Meade K.G., O’Farrelly C. (2019). β-Defensins: Farming the Microbiome for Homeostasis and Health. Front. Immunol..

[61] Raj P.A., Dentino A.R. (2002). Current status of defensins and their role in innate and adaptive immunity. FEMS Microbiol. Lett..

[62] Wong J.H., Xia L., Ng T.B. (2007). A review of defensins of diverse origins. Curr. Protein Pept. Sci..

[63] Mylonakis E., Podsiadlowski L., Muhammed M., Vilcinskas A. (2016). Diversity, evolution and medical applications of insect antimicrobial peptides. Philos. Trans. R. Soc. B Biol. Sci..

[64] Ganz T. (2003). Defensins: Antimicrobial peptides of innate immunity. Nat. Rev. Immunol..

[65] Koehbach J. (2017). Structure-Activity Relationships of Insect Defensins. Front. Chem..

[66] Lee E., Shin A., Kim Y. (2014). Anti-inflammatory activities of cecropin a and its mechanism of action. Arch. Insect Biochem. Physiol..

[67] Wu Y.-L., Xia L.-J., Li J., Zhang F.-C. (2015). CecropinXJ inhibits the proliferation of human gastric cancer BGC823 cells and induces cell death in vitro and in vivo. Int. J. Oncol..

[68] Sun J.S., Xiao S., Carlson J.R. (2018). The diverse small proteins called odorant-binding proteins. Open Biol..

[69] Vogt R.G., Riddiford L.M. (1981). Pheromone binding and inactivation by moth antennae. Nat. Cell Biol..

[70] Zhou J.-J. (2010). Odorant-Binding Proteins in Insects. Vitam. Horm..

[71] Pelosi P., Pisanelli A.M., Baldaccini N.E., Gagliardo A. (1981). Binding of [^3^H]-2-isobutyl-3-methoxypyrazine to cow olfactory mucosa. Chem. Senses.

[72] Pelosi P., Baldaccini N.E., Pisanelli A.M. (1982). Identification of a specific olfactory receptor for 2-isobutyl-3-methoxypyrazine. Biochem. J..

[73] Pelosi P. (1994). Odorant-Binding Proteins. Crit. Rev. Biochem. Mol. Biol..

[74] Tegoni M., Campanacci V., Cambillau C. (2004). Structural aspects of sexual attraction and chemical communication in insects. Trends Biochem. Sci..

[75] Pelosi P., Eiovinella I., Efelicioli A., Dani F.R. (2014). Soluble proteins of chemical communication: An overview across arthropods. Front. Physiol..

[76] Pelosi P., Zhu J., Knoll W. (2018). Odorant-Binding Proteins as Sensing Elements for Odour Monitoring. Sensors.

[77] Bacchini A., Gaetani E., Cavaggioni A. (1992). Pheromone binding proteins of the mouse, *Mus musculus*. Cell. Mol. Life Sci..

[78] Gu T., Huang K., Tian S., Sun Y., Li H., Chen C., Hao D.-J. (2019). Antennal transcriptome analysis and expression profiles of odorant binding proteins in *Clostera restitura*. Comp. Biochem. Physiol. Part D Genom. Proteom..

[79] Campanacci V., Lartigue A., Hällberg B.M., Jones T.A., Giudici-Orticoni M.-T., Tegoni M., Cambillau C. (2003). Moth chemosensory protein exhibits drastic conformational changes and cooperativity on ligand binding. Proc. Natl. Acad. Sci. USA.

[80] Lartigue A., Campanacci V., Roussel A., Larsson A.M., Jones T.A., Tegoni M., Cambillau C. (2002). X-ray Structure and Ligand Binding Study of a Moth Chemosensory Protein. J. Biol. Chem..

[81] Santana K., Galúcio J.M., Da Costa C.H.S., Santana A.R., Carvalho V.D.S., Nascimento L.D.D., Lima A.H.L., Cruz J.N., Alves C.N., Lameira J. (2019). Exploring the Potentiality of Natural Products from Essential Oils as Inhibitors of Odorant-Binding Proteins: A Structure- and Ligand-Based Virtual Screening Approach To Find Novel Mosquito Repellents. ACS Omega.

[82] Pelosi P., Calvello M., Ban L. (2005). Diversity of Odorant-binding Proteins and Chemosensory Proteins in Insects. Chem. Senses.

[83] Scaloni A., Monti M., Angeli S., Pelosi P. (1999). Structural Analysis and Disulfide-Bridge Pairing of Two Odorant-Binding Proteins from *Bombyx mori*. Biochem. Biophys. Res. Commun..

[84] Spinelli S., Lagarde A., Iovinella I., Legrand P., Tegoni M., Pelosi P., Cambillau C. (2012). Crystal structure of Apis mellifera OBP14, a C-minus odorant-binding protein, and its complexes with odorant molecules. Insect Biochem. Mol. Biol..

[85] Zhou J., Zhang G.-A., Huang W., Birkett M.A., Field L.M., Pickett J.A., Pelosi P. (2004). Revisiting the odorant-binding protein LUSH of *Drosophila melanogaster*: Evidence for odour recognition and discrimination. FEBS Lett..

[86] Leal W.S. (2013). Odorant Reception in Insects: Roles of Receptors, Binding Proteins, and Degrading Enzymes. Annu. Rev. Èntomol..

[87] Forêt S., Wanner K.W., Maleszka R. (2007). Chemosensory proteins in the honey bee: Insights from the annotated genome, comparative analyses and expressional profiling. Insect Biochem. Mol. Biol..

[88] Vieira F.G., Sánchez-Gracia A., Rozas J. (2007). Comparative genomic analysis of the odorant-binding protein family in 12 Drosophila genomes: Purifying selection and birth-and-death evolution. Genome Biol..

[89] Liu Y., Sun L., Cao D., Walker W.B., Zhang Y., Wang G. (2015). Identification of candidate olfactory genes in *Leptinotarsa decemlineata* by antennal transcriptome analysis. Front. Ecol. Evol..

[90] Hekmat-Scafe D.S., Scafe C.R., McKinney A.J., Tanouye M.A. (2002). Genome-Wide Analysis of the Odorant-Binding Protein Gene Family in *Drosophila melanogaster*. Genome Res..

[91] McKenna M.P., Hekmat-Scafe D.S., Gaines P., Carlson J.R. (1994). Putative Drosophila pheromone-binding proteins expressed in a subregion of the olfactory system. J. Biol. Chem..

[92] Menuz K., Larter N.K., Park J., Carlson J.R. (2014). An RNA-Seq Screen of the Drosophila Antenna Identifies a Transporter Necessary for Ammonia Detection. PLoS Genet..

[93] Galindo K., Smith D.P. (2001). A large family of divergent Drosophila odorant-binding proteins expressed in gustatory and olfactory sensilla. Genetics.

[94] Jeong Y.T., Shim J., Oh S.R., Yoon H.I., Kim C.H., Moon S.J., Montell C. (2013). An Odorant-Binding Protein Required for Suppression of Sweet Taste by Bitter Chemicals. Neuron.

[95] Shanbhag S., Park S.K., Pikielny C., Steinbrecht R.A. (2001). Gustatory organs of Drosophila melanogaster: Fine structure and expression of the putative odorant-binding protein PBPRP2. Cell Tissue Res..

[96] Pelosi P., Iovinella I., Zhu J., Wang G., Dani F.R. (2017). Beyond chemoreception: Diverse tasks of soluble olfactory proteins in insects. Biol. Rev..

[97] Li S., Picimbon J.-F., Ji S., Kan Y., Chuanling Q., Zhou J.-J., Pelosi P. (2008). Multiple functions of an odorant-binding protein in the mosquito Aedes aegypti. Biochem. Biophys. Res. Commun..

[98] Sirot L.K., Poulson R.L., McKenna M.C., Girnary H., Wolfner M.F., Harrington L.C. (2008). Identity and transfer of male reproductive gland proteins of the dengue vector mosquito, Aedes aegypti: Potential tools for control of female feeding and reproduction. Insect Biochem. Mol. Biol..

[99] Xu J., Baulding J., Palli S.R. (2013). Proteomics of Tribolium castaneum seminal fluid proteins: Identification of an angiotensin-converting enzyme as a key player in regulation of reproduction. J. Proteom..

[100] Sun Y.-L., Huang L.-Q., Pelosi P., Wang C.-Z. (2012). Expression in Antennae and Reproductive Organs Suggests a Dual Role of an Odorant-Binding Protein in Two Sibling Helicoverpa Species. PLoS ONE.

[101] Vieira F.G., Rozas J. (2011). Comparative Genomics of the Odorant-Binding and Chemosensory Protein Gene Families across the Arthropoda: Origin and Evolutionary History of the Chemosensory System. Genome Biol. Evol..

[102] He X., Tzotzos G., Woodcock C., Pickett J.A., Hooper T., Field L.M., Zhou J.-J. (2010). Binding of the General Odorant Binding Protein of Bombyx mori BmorGOBP2 to the Moth Sex Pheromone Components. J. Chem. Ecol..

[103] Vogt R.G., Prestwich G.D., Lerner M.R. (1991). Odorant-binding-protein subfamilies associate with distinct classes of olfactory receptor neurons in insects. J. Neurobiol..

[104] Ziegelberger G. (2008). Redox-Shift of the Pheromone-Binding Protein in the Silkmoth Antheraea Polyphemus. JBIC J. Biol. Inorg. Chem..

[105] Meillour P.N.-L., Joly A., Le Danvic C., Marie A., Zirah S., Cornard J.P. (2019). Binding Specificity of Native Odorant-Binding Protein Isoforms Is Driven by Phosphorylation and O-N-Acetylglucosaminylation in the Pig Sus scrofa. Front. Endocrinol..

[106] Chang H., Liu Y., Yang T., Pelosi P., Dong S., Wang G. (2015). Pheromone binding proteins enhance the sensitivity of olfactory receptors to sex pheromones in *Chilo suppressalis*. Sci. Rep..

[107] Bianchi F., Flisi S., Careri M., Riboni N., Resimini S., Sala A., Conti V., Mattarozzi M., Taddei S., Spadini C. (2019). Vertebrate odorant binding proteins as antimicrobial humoral components of innate immunity for pathogenic microorganisms. PLoS ONE.

[108] Krieger J., Von Nickisch-Rosenegk E., Mameli M., Pelosi P., Breer H. (1996). Binding proteins from the antennae of *Bombyx mori*. Insect Biochem. Mol. Biol..

[109] Zhou J.-J., Robertson G., He X., Dufour S., Hooper A.M., Pickett J.A., Keep N.H., Field L.M. (2009). Characterisation of Bombyx mori Odorant-binding Proteins Reveals that a General Odorant-binding Protein Discriminates Between Sex Pheromone Components. J. Mol. Biol..

[110] Hu P., Gao C., Zong S., Luo Y., Tao J. (2018). Pheromone Binding Protein EhipPBP1 Is Highly Enriched in the Male Antennae of the Seabuckthorn Carpenterworm and Is Binding to Sex Pheromone Components. Front. Physiol..

[111] Pesenti M.E., Spinelli S., Bezirard V., Briand L., Pernollet J.-C., Tegoni M., Cambillau C. (2008). Structural Basis of the Honey Bee PBP Pheromone and pH-induced Conformational Change. J. Mol. Biol..

[112] Yang H., Liu Y.-L., Tao Y.-Y., Yang W., Yang C.-P., Zhang J., Qian L.-Z., Liu H., Wang Z.-Y. (2019). Bioinformatic and biochemical analysis of the key binding sites of the pheromone binding protein of *Cyrtotrachelus buqueti* Guerin-Meneville (Coleoptera: Curculionidea). PeerJ.

[113] Leal W.S., Nikonova L., Peng G. (1999). Disulfide structure of the pheromone binding protein from the silkworm moth, *Bombyx mori*. FEBS Lett..

[114] Zhang Y.-N., Jin J.-Y., Jin R., Xia Y.-H., Zhou J.-J., Deng J.-Y., Dong S.-L. (2013). Differential Expression Patterns in Chemosensory and Non-Chemosensory Tissues of Putative Chemosensory Genes Identified by Transcriptome Analysis of Insect Pest the Purple Stem Borer Sesamia inferens (Walker). PLoS ONE.

[115] De Santis F., François M.-C., Merlin C., Pelletier J., Maïbèche-Coisné M., Conti E., Jacquin-Joly E. (2006). Molecular Cloning and in Situ Expression Patterns of Two New Pheromone-Binding Proteins from the Corn Stemborer *Sesamia nonagrioides*. J. Chem. Ecol..

[116] Vogel H., Heidel A.J., Heckel D.G., Groot A.T. (2010). Transcriptome analysis of the sex pheromone gland of the noctuid moth *Heliothis virescens*. BMC Genom..

[117] Gu S.-H., Zhou J.-J., Wang G.-R., Zhang Y., Guo Y.-Y. (2013). Sex pheromone recognition and immunolocalization of three pheromone binding proteins in the black cutworm moth *Agrotis ipsilon*. Insect Biochem. Mol. Biol..

[118] Forstner M., Gohl T., Breer H., Krieger J. (2006). Candidate pheromone binding proteins of the silkmoth *Bombyx mori*. Invertebr. Neurosci..

[119] Klein U. (1987). Sensillum-lymph proteins from antennal olfactory hairs of the moth *Antheraea polyphemus* (Saturniidae). Insect Biochem..

[120] Pelosi P., Zhou J.-J., Ban L.P., Calvello M. (2006). Soluble proteins in insect chemical communication. Cell. Mol. Life Sci..

[121] Zhang J., Walker W.B., Wang G. (2015). Pheromone Reception in Moths. Prog. Mol. Biol. Transl. Sci..

[122] Yang H., Su T., Yang W., Yang C., Lu L., Chen Z. (2017). The developmental transcriptome of the bamboo snout beetle Cyrtotrachelus buqueti and insights into candidate pheromone-binding proteins. PLoS ONE.

[123] Srygley R.B. (2016). Mormon crickets maximize nutrient intake at the expense of immunity. Physiol. Èntomol..

[124] Alonso-Zarazaga M.A., Barrios H., Borovec R., Bouchard P., Caldara R., Colonnelli E., Gültekin L., Hlaváč P., Korotyaev B., Lyal C.H. (2017). Cooperative Catalogue of Palaeartic Coleoptera Curculionoidea. Monogr. Electrón. SEA.

